# Comparison of left versus right atrial myocardium in patients with sinus rhythm or atrial fibrillation – an assessment of mitochondrial function and microRNA expression

**DOI:** 10.14814/phy2.12124

**Published:** 2014-08-28

**Authors:** Katrine Hordnes Slagsvold, Anne Berit Johnsen, Øivind Rognmo, Morten Høydal, Ulrik Wisløff, Alexander Wahba

**Affiliations:** 1 K.G. Jebsen Center of Exercise in Medicine, Department of Circulation and Medical Imaging, Faculty of Medicine, Norwegian University of Science and Technology, Trondheim, Norway; 2 Department of Cardiothoracic Surgery, St. Olav's Hospital, Trondheim University Hospital, Trondheim, Norway

**Keywords:** Arrhythmia, atrium, microRNA, mitochondria

## Abstract

Several of the cellular alterations involved in atrial fibrillation (AF) may be linked to mitochondrial function and altered microRNA (miR) expression. A majority of studies on human myocardium involve right atrial (RA) tissue only. There are indications that AF may affect the two atria differentially. This study aimed to compare interatrial differences in mitochondrial respiration and miR expression in the RA versus left atrium (LA) within patients with sinus rhythm (SR) and AF. Thirty‐seven patients with AF (*n* = 21) or SR (*n* = 16), undergoing coronary artery bypass surgery and/or heart valve surgery, were included. Myocardial biopsies were obtained from RA and LA appendages. Mitochondrial respiration was assessed in situ in permeabilized myocardium. MiR array and real‐time quantitative polymerase chain reaction were performed to evaluate miR expression. Mitochondrial respiratory rates were similar in RA versus LA. Expression of miR‐100, ‐10b, ‐133a, ‐133b, ‐146a, ‐155, ‐199a‐5p, ‐208b, and ‐30b were different between the atria in both SR and AF patients. In contrast, differential expression was observed between RA versus LA for miR‐93 in patients with SR only, and for miR‐1, ‐125b, ‐142‐5p, ‐208a, and ‐92b within AF patients only. These results indicate that mitochondrial respiratory capacity is similar in the RA and LA of patients with SR and AF. Differences in miR expressional profiles are observed between the RA versus LA in both SR and AF, and several interatrial differences in miR expression diverge between SR and AF. These findings may contribute to the understanding of how AF pathophysiology may affect the two atria differently.

## Abstract (precis)

There are indications that atrial fibrillation (AF) may affect the two atria differentially. We assess interatrial differences in mitochondrial respiration and microRNA expression within patients with AF versus SR. The results of our study indicate that mitochondrial respiratory capacity is similar in the right versus the left atrium of patients with SR and AF. Differences in microRNA expression are observed between the right versus left atrium in both SR and AF, and several interatrial differences in miR expression diverge between SR and AF.

## Introduction

Atrial fibrillation (AF) is prevalent in the increasingly aging population, and is associated with serious complications (Go et al. 2001; Fuster et al. 2011). The underlying pathophysiology of AF is still not fully understood (Mathew et al. 2009). There are indications that AF may affect the two atria differentially, and because the human right atrial (RA) appendage is more accessible for sampling than the left atrial (LA) appendage, investigations of the human LA are particularly scarce (Caballero et al. 2010). Since the majority of studies investigating human tissue are restricted to the RA only, there is limited knowledge regarding interatrial differences in AF and sinus rhythm (SR) in human myocardium.

Despite the central role of mitochondrial respiration in cardiomyocyte function, research on mitochondrial function in AF is limited. Many of the pathophysiological processes implicated in AF are linked to mitochondrial function, including calcium homeostasis (Mihm et al. 2001; Neef et al. 2010; Reilly et al. 2011), formation of reactive oxygen species (ROS) (Mihm et al. 2001), and alterations of oxygen consumption (White et al. 1982). Findings of altered levels of phosphocreatine (Ausma et al. 2000), electron transport chain proteins (Reilly et al. 2011), and differences in mitochondrial distribution (Morillo et al. 1995) further implicate that mitochondria have a role in AF.

MicroRNA (miR) regulates gene expression through inhibition or degradation of mRNA and has been implicated as an important influential element in mitochondrial respiration (Li et al. 2012; Sripada et al. 2012) and cardiac arrhythmias (Luo et al. 2010; Wang et al. 2011), including AF (Shi et al. 2013; Santulli et al. 2014).

We recently presented a report where we compared biatrial mitochondrial function and miR expression between patients with AF versus patients with SR, and found that AF was associated with altered miR expression and elevated maximal mitochondrial respiratory rate as compared to SR (Slagsvold et al. 2014). In this paper, we assess interatrial differences in mitochondrial respiration and miR expression within each of these two patient groups.

## Methods

### Patients

A total of 37 patients with either SR (*n* = 16) or a history of AF (*n* = 21) scheduled for elective isolated coronary artery bypass grafting (CABG) surgery, mitral valve replacement (MVR) surgery, aortic valve replacement (AVR) surgery, or combined CABG/AVR surgery at St. Olav's Hospital, Trondheim University Hospital, Norway were included. Patients with either paroxysmal AF (pAF) (*n* = 11) or chronic AF (cAF) (*n* = 10) were included in the AF group. Blinding of patient characteristics was ensured until completion of data collection and processing. The study conformed to the principles outlined in the Declaration of Helsinki, and was approved by the Regional Committee for Medical Research Ethics of Norway. Written informed consent was obtained from patients prior to inclusion.

### Study design

Standard pre‐ and peroperative procedures of the Department of Cardiothoracic Surgery, St. Olav's Hospital were followed. Thiopental, fentanyl, propofol, and cisatracurium were administered intravenously for anesthetics. Isoflurane was given during pulmonary ventilation, but not during cardiopulmonary bypass (CPB). CPB was performed at mild hypothermia of 32–34°C. Standard St. Thomas (Martindale Pharmaceuticals, Essex, United Kingdom) crystalloid or blood cardioplegia was administered for myocardial protection. Cardiac rhythm was monitored pre‐ and peroperatively by electrocardiography.

### Tissue samples

Biopsies from the RA appendage were obtained during RA cannulation, and biopsies from the LA appendage during CPB. Each biopsy was immediately divided into two; one piece was promptly submerged in an ice‐cold preservation solution for assessment of mitochondrial function, and the other snap‐frozen in liquid nitrogen and kept on −80°C for miR analyses.

### Mitochondrial respiration in situ

Mitochondrial respiration rates were measured *in situ* according to methods thoroughly described by other authors (Veksler et al. 1987; Saks et al. 1998; Zoll et al. 2002; N'Guessan et al. 2004; Kuznetsov et al. 2008). The myocardium was separated from connective tissue under a microscope, and myocardial cell membranes permeabilized by 50 μg/mL saponin for 30 min at 4°C while leaving mitochondrial membranes intact. This was followed by a > 10 min rinsing cycle in pure storage solution (2.77 mmol/L CaK_2_EGTA, 7.23 mmol/L K_2_EGTA (100 nmol/L free Ca^2+^), 6.56 mmol/L MgCl_2_ (1 mmol/L free Mg^2+^), 20 mmol/L taurine, 0.5 mmol/L dithiothreitol (DTT), and 20 mmol/L imidazole 50 mmol/L potassium‐methanesulfonate (CH_3_KO_3_S), 5.7 mmol/L Na_2_ATP, 15 mmol/L phosphocreatine (PCr) (pH 7.1 at 22°C)), and subsequent 10 min rinsing in respiration solution (2.77 mmol/L CaK_2_EGTA, 7.23 mmol/L K_2_EGTA (100 nmol/L free Ca^2+^), 1.38 mmol/L MgCl_2_ (1 mmol/L free Mg^2+^), 20 mmol/L taurine, 0.5 mmol/L dithiothreitol (DTT), and 20 mmol/L imidazole (pH 7.1 at 22°C), 90 mmol/L potassium‐methanesulfonate (CH_3_KO_3_S), 10 mmol/L sodium‐methanesulfonate (CH_3_SO_3_Na), 3 mmol/L K_2_HPO_4_, 10 mmol/L glutamate, 4 mmol/L malate, and 2 mg/mL bovine serum albumin). Mitochondrial respiratory assessment of the permeabilized atrial myocardium was performed by use of a Clark‐type microcathode oxygen electrode (Strathkelvin Instruments, Glasgow, UK) covered by a fluorinated ethylene propylene (FEP) membrane, with tissue submerged in 3 mL respiration solution maintained at 22°C. Assessment of respiration in the presence of respiratory complex I substrates glutamate and malate, without adenosine diphosphate (ADP) (basal respiratory rate, V_0_), was followed by assessment of respiration in the presence of subsaturating 0.1 mmol/L ADP (*V*_ADP_). A volume of 20 mmol/L creatine was supplemented prior to measurement of *V*_creatine_, and subsequently the addition of a saturating 2 mmol/L ADP allowed assessment of maximal respiration rate (*V*_max_) with glutamate and malate as substrates for complex I through IV of the electron transport chain. *V*_succinate_ was measured in the presence of complex II substrate succinate (10 mmol/L) and subsequent complex I inhibition by amytal (1 mmol/L) preceded assessment of complex II (*V*_amytal_). Ascorbate (0.5 mmol/L) and N,N,N’,N’‐tetramethyl‐p‐phenylenediamine (TMPD, 0.5 mmol/L) was added to induce complex IV activity (*V*_ascorbateTMPD_) and before completion irreversible inhibition of complex IV by azide (4 mmol/L) allowed assessment of *V*_azide_. Respiratory rates are given as micromoles O_2_ per minute per gram dry weight myocardial tissue (μmol O_2_/min/g dw). Acceptor control ratio (ACR), as calculated by the ratio *V*_max_/V_0_, estimates degree of coupling between oxidation and phosphorylation. The ratio of *V*_ADP_/*V*_max_ quantifies mitochondrial sensitivity to ADP. Percent increase in respiratory rate after addition of creatine is given (↑RR Cr). The ratio *V*_amytal_/*V*_max_ estimates excess respiration of the cytochrome oxidase complex. Estimation of the apparent constant of Michaelis for ADP was calculated in the absence (^app^K_m_^(ADP−Cr)^) and presence of creatine (^app^K_m_^(ADP+Cr)^) (N'Guessan et al. 2004).

### MicroRNA expression

RNA isolation, miR array and real‐time quantitative polymerase chain reaction (qRT‐PCR) were performed by Exiqon Services, Vedbaek, Denmark. RNA quality was ascertained by use of Agilent 2100 bioanalyzer (Agilent Technologies, Inc., Santa Clara, CA) prior to labeling for miR array by use of Exiqon's miRCURY LNA^™^ microRNA Hi‐Power Labeling Kit, Hy3^™^/Hy5^™^ and subsequent hybridization on the miRCURY LNA^™^ microRNA Array 6th gen (Exiqon, Denmark). Capture probes targeting all human miRs registered in miRBASE 16.0 were applied. Hybridization was performed with a Tecan HS4800^™^ hybridization station (Tecan, Austria). Agilent G2565BA Microarray Scanner System (Agilent Technologies, Inc.) was used to scan the array slides, followed by image analysis with ImaGene^®^ 9 (miRCURY LNA^™^ microRNA Array Analysis Software, Exiqon, Denmark). Background correction of quantified signals was performed prior to normalization by applying the global Lowess (LOcally WEighted Scatterplot Smoothing) regression algorithm. miR RT‐qPCR was performed with miRCURY LNA^™^ Universal RT miRNA PCR pick and mix custom panel, and LightCycler^®^ 480 Real‐Time PCR System (Roche, USA) was used for amplification. PCR was performed for miR‐1, ‐100, ‐106a, ‐106b, ‐10b, ‐125b, ‐133a, ‐133b, 138‐1*, ‐142‐5p, ‐144, ‐146a, ‐155, ‐15b, ‐17, ‐187, ‐18a, ‐18b, ‐191, ‐193a‐3p, ‐199a‐5p, ‐19a, ‐19b, ‐208a, ‐208b, ‐21, ‐23a, ‐25, ‐26a, ‐26b, ‐29b, ‐30a, ‐30b, ‐328, ‐363, ‐451, ‐486‐5p, ‐590‐5p, ‐600, ‐92a, ‐92b, ‐93. Roche LC software was applied for analyses of amplification curves both in determining crossing points (Cp) and melting curves. Algorithms similar to the LinReg software were used to quantify amplification efficiency. Normalization was based on the average of 5 normalization assays detected in all samples. Results of miR expression after RT‐qPCR are presented as normalized crossing point (dCp, refers to the crossing point (Cp) of the specific miR after subtracting the average Cp of the normalization miRs).

### Statistical analysis

SPSS 21.0 for Mac (IBM SPSS Statistics, Chicago, Illinois) was used for statistical analyses. Paired students t‐test was applied for comparison of RA versus LA within each patient. Pearson's Chi^2^ and Fisher's exact test were used for categorical data. A two‐tailed *P*‐value <0.05 was considered significant. Hochberg–Bonferroni‐correction was applied to statistical analyses of miR expression. GraphPad Prism 5 (GraphPad Software, Inc., San Diego, CA) was used for graphics.

## Results

Table 1 provides an overview of clinical data. The groups were comparable with regards to clinical characteristics, with the exception of a statistically significant larger percentage of mitral valve disease in patients with AF as compared to SR. Information on LA diameter was available for 12 patients in SR and 13 in AF and indicated a nonsignificant tendency of LA dilatation in patients with AF. All patients diagnosed with cAF and one of the patients with pAF were in AF on preoperative electrocardiography.

**Table 1.** Patient characteristics and perioperative parameters

	SR (*n* = 16)	AF (*n* = 21)	*P*
Age, μ ± SD	71 ± 8	70 ± 8	ns
Female gender, %	25	24	ns
Aortic valve disease, %	31	43	ns
Mitral valve disease, %	44	76	<0.05
Chronic obstructive pulmonary disease, %	6	5	ns
Coronary artery disease, %	75	81	ns
Diabetes Mellitus, %	13	16	ns
Hypertension, %	50	48	ns
Previous cerebral insult, %	0	5	ns
Previous myocardial infarction, %	31	24	ns
Normal left atrial diameter (≤40 mm), %	83	46	ns
Dilated left atrium (≥41 mm), %	17	54	ns
LV EF, μ ± SD	49 ± 8	52 ± 8	ns
Combined CABG/AVR‐surgery, %	13	24	ns
Isolated AVR‐surgery, %	19	19	ns
Isolated CABG‐surgery, %	63	48	ns
Isolated MVR‐surgery, %	6	10	ns
ACE‐inhibitor/ATII inhibitor, %	44	57	ns
Antiarrhythmic agents, %	6	5	ns
Beta blocker, %	63	76	ns
Calcium antagonist, %	31	24	ns
Diuretics, %	38	48	ns
Digitalis, %	0	5	ns

μ, mean; SD, standard deviation; SR, sinus rhythm; AF, atrial fibrillation; AVR, aortic valve replacement; MVR, mitral valve replacement; CABG, coronary artery bypass graft; LV EF, left ventricular ejection fraction; ACE, angiotensin converting enzyme; ATII, angiotensin II receptor.

### Mitochondrial respiration

**Figure 1.**
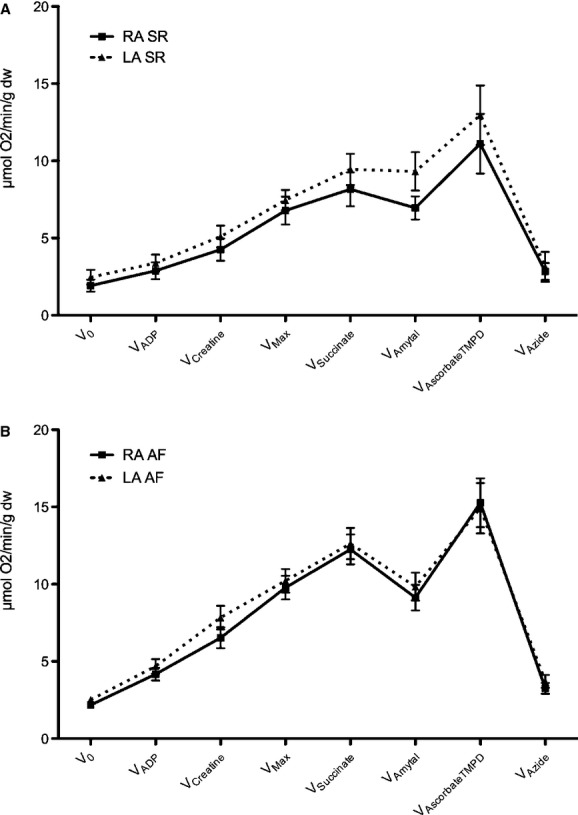
Mitochondrial respiration rates within the left (LA) versus right atrium (RA) of patients with (A) sinus rhythm (SR) and (B) atrial fibrillation (AF). Respiratory rates are given as mean ± SEM (error bars) in μmol O_2_/minute/g dryweight myocardial tissue, and are measured after subsequent addition of the following substrates and inhibitors: V_0_, basal respiration with glutamate and malate as substrates for Complex I of the electron transport chain; *V*_ADP_, respiration in the presence of a subsaturating amount of adenosine diphosphate (ADP); *V*_creatine_, respiration rate after creatine supplement; *V*_max_, maximal respiration rate in the presence of glutamate and malate with a saturating amount of ADP; *V*_succinate_, respiration rate with Complex II substrate succinate; *V*_amytal_, respiration during inhibition of complex I by amytal; *V*_ascorbateTMPD_, respiratory rate with induction of complex IV activity through ascorbate (0.5 mmol/L) and N,N,N’,N’‐tetramethyl‐p‐phenylenediamine (TMPD, 0.5 mmol/L), *V*_azide_, respiration after irreversible inhibition of complex IV by azide.

No significant differences were observed in paired comparison of mitochondrial respiration rates in samples from the RA versus LA within either SR or AF (Fig. 1), nor were there any differences in other parameters of mitochondrial function (Table 2).

**Table 2.** Mitochondrial parameters of the right (RA) versus left atrium (LA) in patients with sinus rhythm (SR) or atrial fibrillation (AF)

	SR			AF		
	LA (*n* = 10)	RA (*n* = 16)	*P*	LA (*n* = 21)	RA (*n* = 21)	*P*
	μ ± SD	μ ± SD		μ ± SD	μ ± SD	
*V*_amytal_/*V*_max_	1.3 ± 0.5	1.1 ± 0.3	ns	1.0 ± 0.3	1.0 ± 0.3	ns
*V*_ADP_/*V*_max_	0.5 ± 0.3	0.4 ± 0.3	ns	0.5 ± 0.1	0.4 ± 0.2	ns
ACR	4.2 ± 2.4	4.9 ± 3.9	ns	4.5 ± 1.7	5.0 ± 1.9	ns
^app^K_m_^(ADP−Cr)^	210 ± 168	194 ± 149	ns	141 ± 77	158 ± 101	ns
^app^K_m_^(ADP+Cr)^	76 ± 80	101 ± 123	ns	43 ± 34	65 ± 39	ns
↑RR Cr	65 ± 50	73 ± 57	ns	69 ± 33	63 ± 35	ns

Data presented as mean ± SD. n, number of patients; SR, sinus rhythm; AF, atrial fibrillation; V_amytal_/V_max_, quantification of excess respiration of the cytochrome oxidase complex; V_ADP_/V_max_, ADP sensitivity ratio; ACR, acceptor control ratio; ^app^K_m_^(ADP−Cr)^ approximate Michaelis‐Menten constant for ADP (μM) in the absence of creatine; ^app^K_m_^(ADP+Cr)^, apparent Michaelis‐Menten constant for ADP (μM) in the presence of creatine; ↑RR Cr, increase in respiration rate after addition of creatine.

### MicroRNA

**Figure 2.**
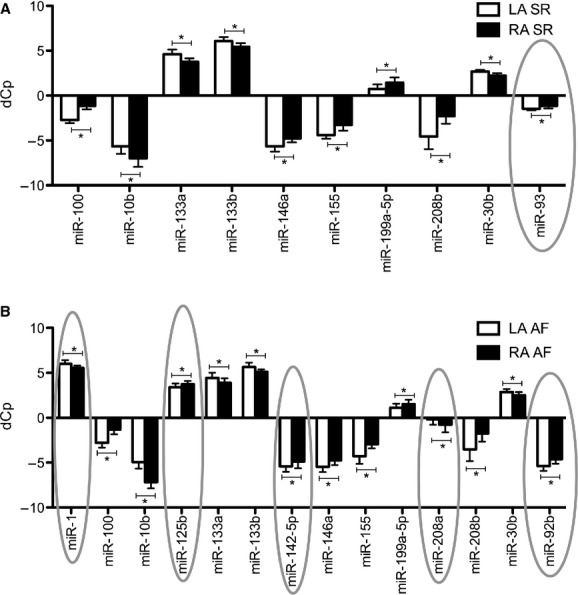
Significant differences in microRNA (miR) expression between the left (LA) versus right atrium (RA) within patients with (A) sinus rhythm (SR) and patients with (B) atrial fibrillation (AF). dCp, normalized crossing point. Circles indicate interatrial differences in miR expression that are found exclusively within either SR or AF patients. Error bars indicate standard deviation. *P < 0.05.

MiR array revealed 75 miRs that were differentially regulated between the LA and RA (data not shown). Array was used as a preliminary explorative study to identify miRs for investigation with qRT‐PCR. qRT‐PCR revealed increased LA expression of miR‐10b, ‐133a, ‐133b, and ‐30b, and decreased expression of miR‐100, ‐146a, ‐155, ‐199a‐5p, and ‐208b as compared to RA in both the SR group and the AF group (Fig. 2).

MiR‐93 displayed decreased expression in the LA as compared to the RA within patients with SR only (Fig. 2A).

In samples from AF patients, increased expression of miR‐1 and ‐208a and decreased expression of miR‐125b, ‐142‐5p, and ‐92b was observed in the LA as compared to the RA in addition to the differences described in both patient groups (Fig. 2B).

## Discussion

Comparison of the LA and RA revealed similar mitochondrial respiratory function in the two atria for patients with SR as well as in patients with AF. Mitochondrial respiration has never previously been compared between the LA and RA of patients with AF, and to our knowledge, only one study has previously assessed mitochondrial function in both human atria, where atrial tissue from two patients without known cardiac disease, and three patients with chronic heart failure were examined, demonstrating similar mitochondrial respiration rates in RA as compared to LA in agreement with our study (Lemieux et al. 2011). These results indicate that, although a difference of interatrial pressures normally exists between the RA and LA, the difference in workload does not appear to have detectable impact on mitochondrial respiratory capacity. These findings are of interest because they may increase our understanding of how results obtained from one myocardial location may relate to another, especially due to the increased attainability of RA appendage tissue in investigations involving the human myocardium (Ausma et al. 2000; Mihm et al. 2001; Kim et al. 2005; Reilly et al. 2011). It could have been expected that mitochondrial respiratory capacity would have been increased in the LA as compared to the RA of patients with AF due to an increased frequency of elevated LA pressure in this patient group (Stefanadis et al. 2001). However, our study does not support this notion. This may, however, depend on the extent of LA dilatation, and it must be kept in mind that our patient population did not reveal significant differences in LA diameter between the patient populations in AF versus SR; thus it cannot be excluded that the results may differ in other patient populations with greater differences in LA diameter.

We observed a number of interatrial differences in miR expression that are common to both patient groups, whereas there are other interatrial differences in miR expression that differ between SR and AF patients. Differences in miR expression between LA and RA that are found in both patients groups may be ascribed to anatomic and physiological differences in the RA as compared to the LA (increased LA expression of miR‐10b, ‐133a, ‐133b, and ‐30b, and decreased expression of miR‐100, ‐146a, ‐155, ‐199a‐5p, – 208b, as compared to RA).

The reduced expression of miR‐93 in the LA versus RA of patients with normal SR may also be related to a difference of anatomical and physiological regions. It has been demonstrated that miR‐93 has proangiogenic effects, promotes perfusion recovery after ischemia, and is associated with improved cell survival after hypoxia and serum starvation in endothelial and skeletal muscle cells (Hazarika et al. 2013).

Interatrial differences found in AF patients only include increased LA expression of miR‐1 and ‐208a and decreased expression of miR‐125b, ‐142‐5p, and ‐92b as compared to the RA. Whereas electrophysiological mapping has indicated that myocardial areas in both the RA and LA may be responsible for activating atrial premature complexes in patients with AF (Natale et al. 2000), there are indications that electrical remodeling is altered differentially in RA as compared to LA in patients with AF (Caballero et al. 2010). Overexpression of miR‐1 has been implicated in promoting arrhythmogenesis (Terentyev et al. 2009). Both overexpression and underexpression of the cardiospecific miR‐208a have been demonstrated to induce cardiac arrhythmia by disturbing atrial electrophysiology in mice (Callis et al. 2009). Blocking cardiac miR‐208a expression prevented cardiac remodeling and dysfunction and promoted health and survival of rats in a study of chronic hypertension and diastolic heart failure (Montgomery et al. 2011). In our previous report where we compared atrial miR expression between patients with SR versus AF, miR‐208a expression was significantly lower in AF patients as compared to patients with SR in both atria (Slagsvold et al. 2014). MiR‐125b is upregulated under hypoxia and upon stimulation with VEGF in skeletal muscle (Muramatsu et al. 2013). It has been demonstrated that miR‐142‐5p regulates genes involved in cell cycle control and increases proliferation and DNA synthesis in vascular smooth muscle cells of rats (Kee et al. 2013). Reduced myocardial expression of miR‐92b has been demonstrated in mice with experimentally induced heart failure (Dirkx et al. 2013).

Investigations of miR expression in human myocardium are still at an early stage, and this study provides information of myocardial miR expression that is strictly descriptive in nature. Moreover, these data do not provide information as to whether any direct link exists between miR expression and mitochondrial function. Many unknown factors still need clarification, and even associative observations are valuable in the process of obtaining an understanding of miR in cardiovascular pathophysiology. Although direct comparison of the results are precluded due to differences in patient characteristics, we identified two other studies reporting comparative assessment of miR expression in the LA versus RA, one investigating patients with AF and valvular heart disease (Cooley et al. 2012), and the other patients with rheumatic mitral valve disease and either SR or AF (Liu et al. 2014). It is evident that continued research is necessary to establish representativeness of miR expression for specific patient populations, especially due to the common occurrence of concurrent heart disease in patients with AF.

### Study limitations

The atrial appendage is selected for investigation as it constitutes the most accessible location for sampling human myocardial tissue. Whether AF‐related alterations on the cellular level prevail throughout the atrium has not yet been assessed in humans, thus it has not been ascertained that tissue from the atrial appendage is representative to the remaining atrial myocardium of patients. However, it has been demonstrated that structural AF‐related alterations in the atrial appendages of goats corresponded to that of the remaining atrial myocardium (Ausma et al. 1997).

Due to the frequent coexistence of cardiac disease in AF, distinguishing AF‐related alterations from those of concomitant heart disease presents a challenge in investigations of AF. Although the clinical characteristics of the patients groups in our study were comparable in most aspects, there was a difference in prevalence of mitral valve disease. Although investigations of “lone” AF have been demonstrated in animal models (Morillo et al. 1995; Ausma et al. 1997), a potential interference of comorbidity cannot be excluded in our clinical study and further research is required to verify reproducibility.

Our investigation provides a description of associative alterations in miR expression and mitochondrial function. Further investigations are required to explore causal relations between mitochondrial respiration, miR, and AF.

## Conclusion

Mitochondrial respiration is similar between the RA and LA in patients with normal SR as well as in AF. Interatrial differences in miR expression are observed within both patients with SR and patients with AF, and the interatrial differences in miR expression profiles diverge between patients with normal SR and patients with AF.

## Acknowledgments

The authors are particularly grateful to the Department of Cardiothoracic surgery, St. Olavs Hospital, Trondheim University Hospital, Norway, for providing tissue samples.

## Conflict of Interest

None declared.
